# Comprehensive Investigation on Ginsenosides in Different Parts of a Garden-Cultivated Ginseng Root and Rhizome

**DOI:** 10.3390/molecules26061696

**Published:** 2021-03-18

**Authors:** Junqian Pan, Wei Zheng, Xu Pang, Jie Zhang, Xiaojuan Chen, Ming Yuan, Kate Yu, Baolin Guo, Baiping Ma

**Affiliations:** 1 Beijing Institute of Radiation Medicine, No. 27 Taiping Road, Beijing 100850, China; panjunqian123@163.com (J.P.); zhengweixxy@163.com (W.Z.); pangxu320@163.com (X.P.); zhangjie061003@163.com (J.Z.); chenxj626@163.com (X.C.); 2 Waters Technologies (Shanghai) Limited, Shanghai 201206, China; Ming_Yuan@waters.com (M.Y.); kate_yu@waters.com (K.Y.); 3 Institute of Medicinal Plant Development, Chinese Academy of Medical Sciences and Peking Union Medical College, No. 151 Malianwa North Road, Beijing 100193, China; guobaolin010@163.com

**Keywords:** cultivated ginseng root, ginsenosides, UHPLC-Q-TOF/MS, UHPLC-CAD, fibrous root

## Abstract

Background: Ginseng is widely used as herb or food. Different parts of ginseng have diverse usages. However, the comprehensive analysis on the ginsenosides in different parts of ginseng root is scarce. Methods: An ultra-high-performance liquid chromatography-quadrupole time-of-flight mass spectrometry (UHPLC-Q-TOF/MS) combined with UNIFI informatics platform and ultra-high-performance liquid chromatography-charged aerosol detection (UHPLC-CAD) were employed to evaluate the different parts of cultivated ginseng root. Results: 105 ginsenosides including 16 new compounds were identified or tentatively characterized. 22 potential chemical markers were identified, 20, 17, and 19 for main root (MR) and fibrous root (FR), main root (MR) and branch root (BR), and main root (MR) and rhizome (RH), respectively. The relative contents of Re, Rb_1_, 20(R)-Rh_1_, Rd, and Rf were highest in FR. The relative content of Rg_1_ was highest in RH. The total relative content of pharmacopoeia indicators Rg_1_, Re, and Rb_1_ was highest in FR. Conclusion: The differences among these parts were the compositions and relative contents of ginsenosides. Under our research conditions, the peak area ratio of Rg_1_ and Re could distinguish the MR and FR samples. Fibrous roots showed rich ingredients and high ginsenosides contents which should be further utilized.

## 1. Introduction

Ginseng, *Panax ginseng* Meyer of the Araliaceae family, is the king of herbs in the Orient, and its root has been widely used as a constituent of traditional medicine in China and Korea [1]. The major constituents of ginseng root are ginsenosides with various pharmacological properties, such as antitumor, enhanced immune system, antidiabetes, antifatigue, anti-oxidative, and anti-aging effects [2,3,4,5,6]. At present, ginseng is used not only as a therapeutic medicinal herb but also as a health supplement available on the market to promote longevity and adjust the balance of the human body. Public use of ginseng in the food field continues to grow. The quality of ginseng roots in the food industry and herbal markets must be evaluated and controlled. Nowadays, wild ginseng is rarely available, and the types of ginseng on the market are mostly collected from farms cultivating ginseng in fields. Ginseng has been developed as a valuable industrial crop and is now widely used worldwide [7]. The quality of ginseng root varies from different cultivation environments/areas, cultivation ages and “paozhi”. For example, ginsenosides Rg_5_, Rh_4_, Rk_1_, Rs_4_, F_4_, and 20(S)-Rg_3_ were found to be the quality control markers in different processing methods in distinguishing red ginseng from white ginseng [8]. The metabolic profiles of root, leaf, flower bud, berry, and seed of ginseng were also investigated [9,10]. However, the comprehensive analysis and research on the chemical constituents of different parts of ginseng root are scarce.

Ginseng root is further divided into main root (MR), branch root (BR), rhizome (RH), and fibrous root (FR). The different parts of *P. ginseng* root have diverse tradition medicine uses. The main root is generally used in Chinese medical clinics and traditional Chinese proprietary medicines and it was used as slice. The rhizome may cause the vomits, so it will be cut off before make slice. The fibrous root and branch root of ginseng root are the residual products of ginseng slice and it is used for the manufacturing of ginsenosides and related preparations, whereas the fibrous and branch roots are often ground to yield powder as health food. Several reports showed that the contents of total saponins in ginseng root were ranked as follows: rhizome > branch root > main root [11,12]. Few reports are available regarding the chemical constituents of different parts of the ginseng root. Therefore, an in-depth and comprehensive study on the ginsenosides of the different parts of ginseng root must be conducted to investigate. The constituents of such parts will provide the material basis reference for further utilization.

In recent years, ultra-high-performance liquid chromatography-quadrupole time-of-flight mass spectrometry (UHPLC-Q-TOF/MS) has become a powerful tool for the rapid separation and identification of active components in traditional Chinese medicines due to its advantages of high resolution and sensitivity. In addition, UNIFI, the automated data processing software, is an integrated informatics platform that can incorporate scientific library into a streamlined workflow to identify chemical components from complex raw data [13]. The combination of UHPLC separation, Q-TOF/MS detection, and UNIFI platform has been frequently applied in the characterization of chemical constituents of herbs [14,15]. Charged aerosol detector (CAD) has become a valuable tool for detecting substances with no ultraviolet absorption or only end absorption, such as saponins. This detector has some advantages, such as broad linearity response range, high sensitivity and reproducibility, the signal response consistency is independent of chemical structures, and simple operation [16,17]. The CAD detector coupled with UHPLC for a semiquantitative or quantitative analysis has certain advantages.

A comprehensive analysis on the chemical constituents of ginseng root based on UHPLC-Q-TOF/MS and UHPLC-CAD was performed in this study to evaluate the differences of ginsenosides among MR, BR, RH, and FR of cultivated ginseng root. The UHPLC-Q-TOF/MS combined with the UNIFI informatics platform was used to develop a multicomponent identification workflow for the analysis of extracts from four parts of cultivated ginseng root. Principal component analysis (PCA) and orthogonal partial least squares discrimination analysis (OPLS-DA) was used to profile diverse classes of metabolites of four parts from ginseng root. The UHPLC-CAD data were processed by histogram to intuitively see the differences of ginsenoside compounds in different parts of cultivated ginseng root. The study in this research comparatively analyzes the phytochemicals of different parts of ginseng root for the first time and finds out the similarities and differences among them. These results will support the further research and exploration of potential applications of ginseng root.

## 2. Results and Discussion

### 2.1. Identification of Components from Four Different Parts of the Ginseng Root Based on the UNIFI Platform

**Figure 1:**
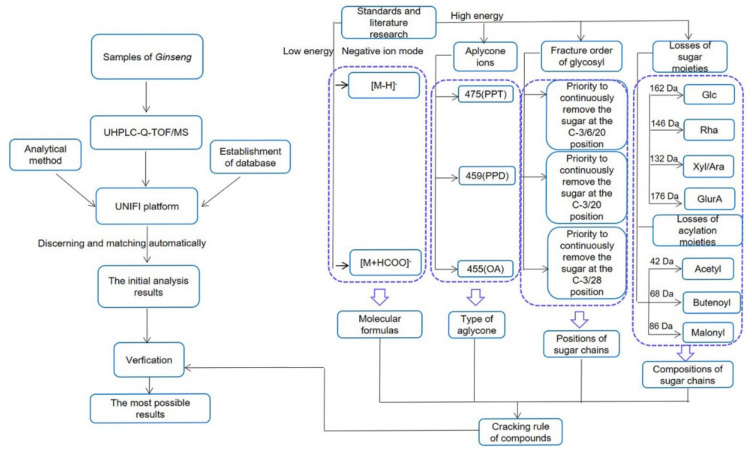
Workflow of the rapid characterization of ginsenoside in *P. ginseng* by UHPLC-Q-TOF/MS combined with the UNIFI informatics platform.

According to the literature [18], the ginsenosides in *P. ginseng* are generally divided into several types according to the aglycone moieties: the protopanaxadiol (PPD) type with sugar moieties attached to C-3 and/or C-20, the protopanaxatriol (PPT) type with sugar moieties at C-3, C-6, and/or C-20, the oleanolic acid type (OA), and other PPD and PPT derivatives. In combination with the fragmentation behavior of the reference standards and some studies [7,8,19,20,21,22], we deduced the fragmentation rule of the ginsenoside in ginseng; we also proposed a strategy for characterization and identification of compounds in *P. ginseng* by using the workflow of UHPLC-Q-TOF/MS combined with UNIFI informatics platform (Figure 1).

The fragmentation rule of ginsenoside in *P. ginseng* is deducted as follows: for low CE in the ESI^−^ mode, the common adduct ions, such as [M + HCOO]^−^ and deprotonated ions [M − H]^−^, were often observed, which would allow us to determine the molecular mass and formula of the compounds. The backbone of a compound could be rapidly assigned by the abundant aglycone ions in the high CE channel of the ESI^−^. The characteristic fragment ion at *m/z* 475, 459, and 455 correspond to the PPT, PPD, and OA type aglycones, respectively. The positions of the glycosyl chains could be readily determined by the characteristic fragment ions in the negative ion mode.

The characteristic sugar fragments can be found by sequential losses of 162 Da (−Glc), and/or 146 Da (−Rha), and/or 132 Da (−Xyl/Ara), and/or 176 Da (−GlurA). Fragmentation can be conducted in three possible ways when the hydroxy groups on the sugar were replaced by acetyl (Ace), butenoyl (But), or malonyl (Mal) groups, which would be accompanied by the losses of 42 Da (−Ace), 68 Da (−But), 44 Da (−CO_2_), and 86 Da (−Mal). Sugar chains first loss the acylation moieties and then the glycosyls. After data processing, all discovered components using UNIFI were further verified.

**Figure 2:**
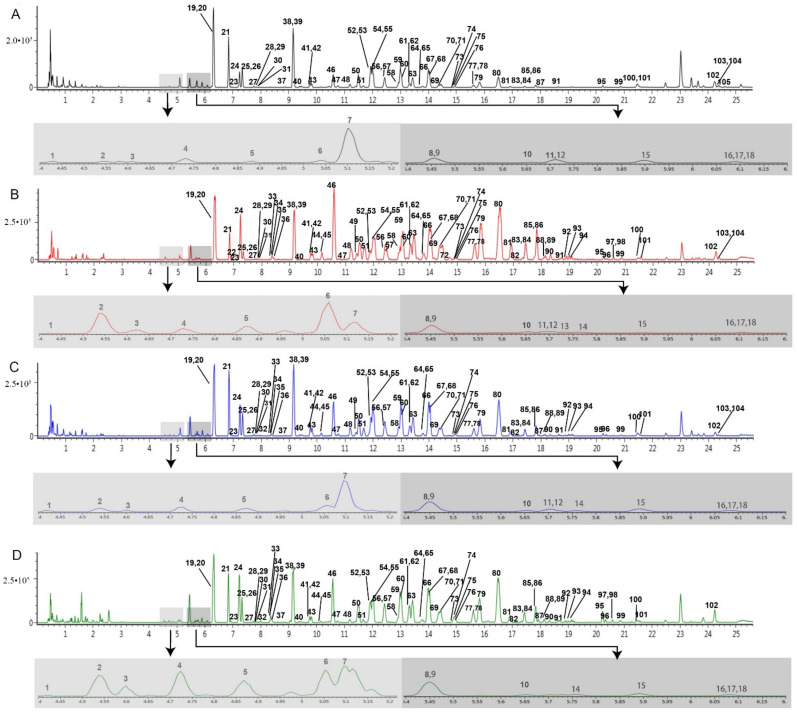
BPI chromatograms of the different parts of the *P. ginseng* root in the negative mode analyzed by UHPLC-Q-TOF/MS^E^. (**A**) main root (MR), (**B**) fibrous root (FR), (**C**) branch root (BR), (**D**) rhizome (RH). Light gray represents a partial enlarged view of the chromatogram with the retention time from 4.4 to 5.2 min, and dark gray represents a partial enlarged view of the chromatogram with the retention time from 5.4 to 6.2 min.

Accordingly, 105 ginsenosides were identified or tentatively characterized in the ESI^−^ modes from MR, BR, FR, and RH (Table 1). The base peak ion (BPI) chromatograms are shown in Figure 2. In the four different parts of the ginseng root, these compounds almost shared constituents, including 57 PPD type saponins, 31 PPT type saponins, 4 OA type saponins, and 13 other compounds. The chemical structures of these compounds were summarized in Table S1. Among them, 83, 101, 99, and 96 ginsenosides were tentatively characterized in the MR, FR, BR, and RH, respectively. This result clearly suggests that these parts could be used as raw materials for the manufacture of ginsenoside-based products.

The compounds were tentatively assigned by matching the molecular formulas and diagnostic fragment ions with those of the published known ginsenosides and the reference standards on the basis of UNIFI.

For example, peaks 5, 7–15, 17–29, 31–33, 36–37, 39–41, 43, 46, and 47 exhibited fragment ions at *m/z* 475 in the high energy of ESI^−^ corresponding to the PPT aglycone moiety, thereby suggesting that they were the PPT-type ginsenosides. Peak 8, 10, and 14 had the same deprotonated molecular ion [M − H]^−^ at *m/z* 931 and an adduct ion [M + HCOO]^−^ at *m/z* 977 in the low energy of ESI^−^ and fragment ions at *m/z* 799 [M − H − 132]^−^ and 637 [M − H − 132 − Glc]^−^ and 475 [M − H − 132 − 2Glc]^−^ in the high energy of ESI^−^, thereby suggesting that they were a pair of isomers. In comparison with the reference standard, peak 14 was assigned to be Notoginsenoside R_1_. Meanwhile, in comparison with literature [7,19], peaks 8 and 10 were tentatively assigned to be Ginsenoside Re_4_ or its isomers. Peaks 7, 9, 11, 15, 18, 27, 33, 36, and 37 should be in each pair of isomers. Peaks 7, 11, and 15 were tentatively identified as Ginsenoside Re_1_/Ginsenoside Re_2_/Ginsenoside Re_3_ or their isomers. Peak 9 was identified as 20-O-Glucosylginsenoside Rf. Peaks 18, 27, 33, 36, and 37 were tentatively identified as Vinaginsenoside R_4_/Notoginsenoside R_3_/Notoginsenoside R_6_/Notoginsenoside M/Notoginsenoside N or its isomers compared with literature [7,19,23]. This study matched the accurate masses and the fragment ions with those of previous studies [7,19,25,27]; peaks 21, 23, 25, 32, and 41 were tentatively assigned to be 6′-O-Acetyl-ginsenoside Rg_1_, Yesanchinoside D, or its isomer; peaks 22 and 24 were tentatively assigned to be 6′′′-O-Acetyl-ginsenoside Re or its isomer; peak 29 was assigned to be Ginsenoside Rs_4_ or Ginsenoside Rs_5_; they all produced fragment ions [M − H − Ace]^−^ and were acetylated ginsenosides.

Peaks 38, 42, 44–45, 48–52, 54–74, 76–94, 96–101, and 103–104 exhibited the fragment ions at *m/z* 459 in the high energy of ESI^−^ corresponding to the PPD aglycone moiety, thereby suggesting that they were the PPD-type ginsenosides. This study takes peaks 79 and 85 as examples. Both peaks had the same protonated ion [M + HCOO]^−^ at *m/z* 991 and the same fragment ions at *m/z* 783 [M − H − Glc]^−^, 621 [M − H − 2Glc]^−^, and 459 [M − H − 3Glc]^−^ in ESI^−^. As a pair of isomers, peak 79 was identified as ginsenoside Rd compared with the standard; peak 85 was tentatively identified as Gypenoside XVII because it was matched with the characteristic MS fragmentation pattern of Gypenoside XVII reported in literature [20]. Peaks 38, 45, 55, 60, 68, 78, 84, and 88 all produced fragment ions [M − H − Mal]^−^ and were malonylated ginsenosides by matching the accurate masses and the fragment ions with those of previous studies; peaks 96, 91, 94, 98, and 87 produced fragment ions [M − H − But]^−^ and were butenoylated ginsenosides; peaks 44, 51, 52, 54, 58–59, 61, 64, 66–67, 69–74, 76–77, 80–83, 86, 89–90, 93, 99, and 101 produced fragment ions [M − H − Ace]^−^ and were acetylated ginsenosides.

Peaks 30, 53, 75, and 95 exhibited fragment ions at *m/z* 455 in the high energy of ESI^−^ corresponding to the OA aglycone moiety, thereby suggesting that they were the OA-type ginsenosides. Peak 30 showed a deprotonated molecular ion [M − H]^−^ at *m/z* 925 in the low energy of ESI^−^, thereby suggesting that the molecular formula was C_47_H_74_O_18_. In the high energy of ESI^−^, the fragment ions [M − H − Glc]^−^ at *m/z* 763, [M − H − Glc − Xyl]^−^ at *m/z* 631, and [M − H − Glc − Xyl − GlurA]^−^ at *m/z* 455 could be attributed to the successive loss of the Glc, Xyl, and GlurA groups. Finally, peak 30 was assigned to be Pseudoginsenoside Rt_1_ [7,28]. Peaks 75 and 95 exhibited the same deprotonated molecular ion [M − H]^−^ at *m/z* 793 in the low energy of ESI^−^, thereby suggesting that the molecular formula was C_42_H_66_O_14_ and the same fragment ions [M − H − Glc]^−^ at *m/z* 631 and [M − H − Glc − GlurA]^−^ at *m/z* 455 in the high energy of ESI^−^. The fragment ions could be attributed to the loss of the Glc and GlurA groups. Finally, peaks 75 and 95 were tentatively assigned to be Chikusetsusaponin Iva or its isomer [26].

In this study, we have tentatively identified 16 new compounds (peaks 2, 6, 64, 66, 70–74, 76–77, 82–83, 90, 93, and 95) from MR, BR, FR, and RH on the basis of the result of UNIFI and literatures. For example, peaks 51, 58, 66, 70, 72, and 73 showed the same molecular formula (C_60_H_100_O_27_) and fragment ions at *m/z* 1209 [M − H − Ace]^−^, *m/z* 1077 [M − H − Ace − Xyl]^−^, *m/z* 945 [M − H − Ace − 2Xyl]^−^, *m/z* 783 [M − H – Ace − 2Xyl − glc]^−^, *m/z* 621 [M − H − Ace − 2Xyl − 2glc]^−^, and *m/z* 459 [M − H − Ace − 2Xyl − 3glc]^−^ in the high energy of ESI^−^ and they were a pair of isomers. Based on the literatures, we found that only two compounds are present, namely, Ginsenoside Ra_5_ and (3β,12β)-3-[[2-O-(6-O-Acetyl-β-D-glucopyranosyl)-β-D-glucopyranosyl]oxy]-12-hydroxydammar-24-en-20-yl O-β-D-xylopyranosyl-(1→2)-O-α-L-arabinopyranosyl-(1→6)-β-D-glucopyranoside in accordance with the mass spectral fragmentation rule of these peaks in this experiment; thus, the other four compounds were tentatively new. In the same way, two new compounds were tentatively identified from the isomers of peaks 54, 61, 64, and 74. Two new compounds were tentatively identified from the isomers of peaks 59, 67, 69, 71, and 76. One new compound was tentatively identified from the isomers of peaks 75 and 95. One new compound was tentatively identified from the isomers of peaks 80, 81, 86, 89, and 93. Peaks 83 and 90 were new compounds that have not been searched in the literature and databases. The high collision energy ESI-MS spectra of some representative compounds in each pair of isomers, such as peaks 51, 54, 59, 75, 80, and 83, are shown in Figure S1.

This research is the first to study the comprehensive screening analysis of the different parts of the cultivated ginseng root by using UHPLC-Q-TOF/MS combined with the UNIFI platform. This comprehensive and unique phytochemical profile study revealed the structural diversity of secondary metabolites and the similar patterns in the different parts of ginseng root. Moreover, this study could provide systematic data for clarifying the chemical composition of ginseng root.

### 2.2. Discrimination of Different Parts of P. ginseng Root by PCA and OPLS-DA Analysis

**Figure 3:**
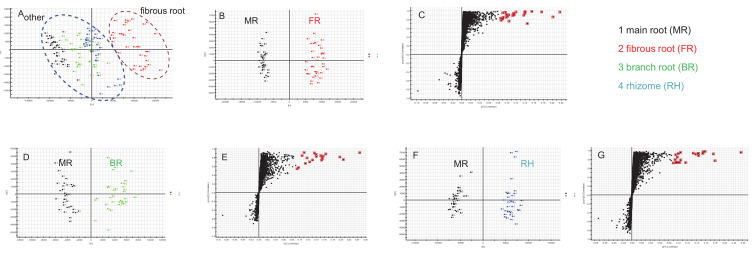
PCA of four different parts of *P. ginseng* root (**A**), OPLS-DA/S plot of MR and FR (**B**,**C**), MR and BR (**D**,**E**), and MR and RH (**F**,**G**) samples in ESI^−^ mode.

Multivariate statistical methods are applied to the analysis of metabolite data to discriminate and classify the different parts of ginseng root and identify marker compounds. First, the obtained multivariate dataset of 144 batches of samples that contained the same amount of MR, BR, RH, and FR was analyzed by PCA. The PCA 2D plots of the samples from four parts of ginseng root groups were easily classified into two clusters according to their common spectral characteristics (Figure 3A). An obvious difference can be observed between FR and other parts, and the FR groups were separated. Figure 3A (right to left) shows a transition trend of FR, RH, BR, and MR.

Aiming at evaluating the differences of the different parts (MR and FR, MR and BR, and MR and RH) of *P. ginseng* root, OPLS-DA score plot and S plot were obtained to understand which variables are responsible for this sample separation. The OPLS-DA plotting achieved maximum separation between different groups (panels B, D and F of Figure 3). S plots were then created to explore the potential chemical markers that contributed to the differences (panels C, E and G of Figure 3). On the basis of the VIP values (VIP > 8.5) from univariate statistical analysis, a total of 22 robust known chemical markers between MR and FR, MR and BR, and MR and RH groups of *P. ginseng* root were marked and listed (Table 2) and there were significant differences in the contents of them. (1) For MR and FR samples, there were 20 potential chemical markers, including 4 PPT-type and 16 PPD-type saponins, with higher contents in FR samples. (2) For MR and BR samples, there were 17 potential chemical markers, including 4 PPT-type and 13 PPD-type saponins, with higher contents in BR samples. (3) For MR and RH samples, there were 19 potential chemical markers, including 4 PPT-type, 13 PPD-type saponins and 2 OA-type (ginsenoside Ro and chikusetsusaponin Iva), with higher contents in RH samples.

According to the reports [34,40], ginsenosides in FR were different from MR and BR in America ginseng and notoginseng. From this point of view, the results in this study are similar to America ginseng and notoginseng. A clear separation from the other parts appeared for FR. Therefore, FR might be differently used from the other parts. In addition, many differential monomeric ginsenosides exist in FR. In future research, some studies could be performed on the pharmacological activities and the relationship between the potential markers, and the effects should be established.

### 2.3. Distribution of Ginsenosides from the Different Parts of P. ginseng Root

The semiquantitative analysis of ginsenosides in different parts of cultivated *P. ginseng* root is still obscured to date. A poor correlation exists between mass spectral response and chemical composition. Accordingly, the data from UHPLC-CAD were used to communicate additional penetrating understanding of the distribution of compounds with a high content in different parts of cultivated *P. ginseng* root, especially pharmacopoeia indicators, such as ginsenosides Rg_1_, Re, and Rb_1_. The separation effects on the ginseng samples of Waters ACQUITY™ HSS T3, Acclaim RSLC PolarAdvantage, and Phenomenex C18 were compared. Rg_1_ and Re could not be separated by Waters ACQUITY™ HSS T3. Although Rg_1_ and Re could be separated by Acclaim RSLC PolarAdvantage column, the separation effect of the other components was worse than that of Phenomenex C18 column. Therefore, the Phenomenex C18 column can be used to separate the components of ginseng root in the Thermo vanquish UHPLC-CAD system.

**Figure 4:**
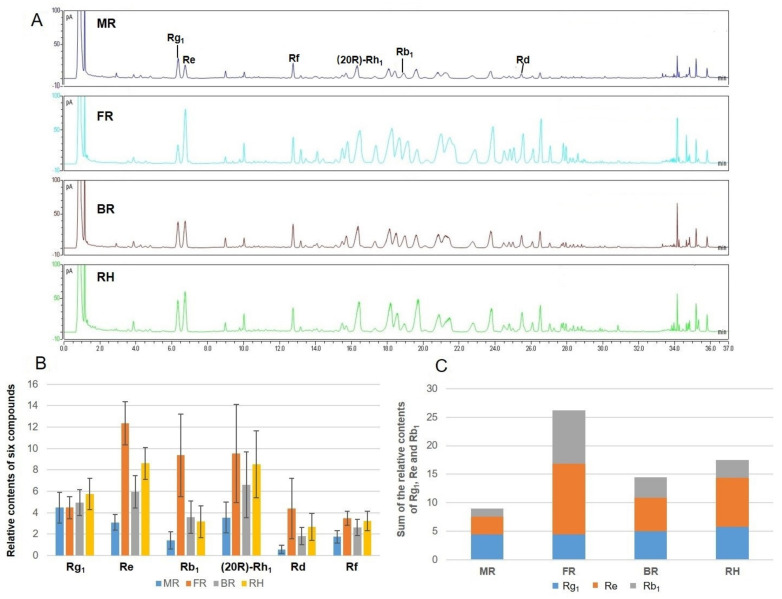
Chromatograms of the different parts of ginseng root (MR main root, FR fibrous root, BR branch root, and RH rhizome) analyzed by UHPLC-CAD and column chart of relative contents of main components. (**A**) liquid chromatograms of different parts of ginseng root; (**B**) relative contents of six components of the different parts of ginseng root; and (**C**) sum of the peak areas of pharmacopoeia indicators Rg_1_, Re, and Rb_1._

Compounds with representative structure, different polarity, high relative content and good pharmacological activity and resolution were selected as the object of relative content analysis in this study. Six main compounds, namely, Rg_1_, Re, Rd, Rb_1_, Rf, and (20R)-Rh_1_, were compared with the existing reference standards. The area percentage method was used to calculate the percentage of the peak area of each compound in the sum of all peak areas, as the evaluation index of relative content. Figure 4A shows the liquid chromatograms of different parts of ginseng root. The FR had more abundant components compared with the other three parts. The contents of most saponins in BR, RH, and FR were higher than those in MR.

The relative contents of the six components in the different parts of 144 ginseng root samples were analyzed (Figure 4B). The results indicated that except for Rg_1_, the relative contents of Re, Rb_1_, (20R)-Rh_1_, Rd, and Rf were the highest in FR, followed by RH, BR, and MR. The peak areas of Re, Rb_1_, (20R)-Rh_1_, Rd, and Rf in FR were 1.43–4, 2.62–6.65, 1.12–2.69, 1.64–7.67, and 1.07–1.98 times than those of the other parts, respectively. The relative content of Rg_1_ in RH was the highest, followed by BR and FR. The peak area of Rg_1_ in RH was 1.16–1.29 times than those of the other parts. The sum of the peak areas of pharmacopoeia indicators Rg_1_, Re, and Rb_1_ in FR was 1.53–3.25 times than those of the other parts (Figure 4C). Ginseng root has two types, with fibrous root or no fibrous root. Our results explain the reason that the content of ginseng with fibrous root is qualified in the market. Under our research conditions, we also found some interesting results. The range of Rg_1_/Re was from 0.19 to 0.64 in FR samples, while the range of Rg_1_/Re was from 0.75 to 2.00 in MR samples. The peak area ratio of Rg_1_ and Re might be the marker of MR and FR samples. This idea laid a theoretical foundation for strengthening the comprehensive utilization of various parts of the roots of garden ginseng in the future.

## 3. Materials and Methods

### 3.1. Materials and Reagents

Thirty-six batches of representative cultivated ginseng root were collected or purchased from different cultivation areas in Jilin, Liaoning, and Heilongjiang Provinces, the main source of ginseng in China. A detailed sample list is provided in Table S2. The identity of all samples was confirmed by Prof. Bao-lin Guo of the Institute of Medicinal Plant Development, Beijing, China. Each whole ginseng root was divided into four parts: main root (MR), branch root (BR), rhizome (RH), and fibrous root (FR). One hundred forty-four different parts samples of ginseng root are present.

Ginsenosides Rb_1_, Re, Rg_1_, Rd, Rc, Rf, Ro, Rg_2_, 20(R)-Rh_1_, 20(S)-Rh_2_, 20(R)-Rh_2_, 20(S)-Rg_3_, and 20(R)-Rg_3_ and notoginsenosides R_1_ and R_2_ were isolated in our laboratory and identified by spectroscopic data. The purities of these standards were better than 98% by the HPLC analysis. All samples were stored at 4 °C before use.

Acetonitrile (HPLC grade) was purchased from Fisher Scientific Co. (Loughborough, UK). Distilled water was purchased from Watsons (Guangzhou, China). Formic acid (MS grade) was purchased from Thermo Fisher Scientific Co. Ltd. (Waltham, MA, USA). The other reagents were commercially obtained in analytical purity (Beijing, China).

### 3.2. Sample Preparation and Extraction

All the samples were separately dried, ground, and sieved (Chinese National Standard Sieve 3, R40/3 Series) to obtain the homogeneous powder. An aliquot of 0.2 g accurately weighed fine powder (<40 mesh) of each sample was soaked in a 10 mL centrifuge tube containing 3 mL of 70% (*v*/*v*) methanol, tightly plugged, shaken, weighed. After sonication for 30 min, the solutions were cooled to room temperature and made up for weight loss with 70% aqueous MeOH. All the solutions were filtered through a 0.22 μm filter membrane before analysis.

### 3.3. UHPLC-Q-TOF/MS and UHPLC-CAD Analysis

A UHPLC-Q-TOF/MS analysis was performed on a Waters ACQUITY I-Class system (Waters Corporation, Milford, MA, USA) coupled with a VION-IMS-QTOF system (Waters Corporation, Wilmslow, UK). A Waters ACQUITY™ UPLC HSS T3 column (100 × 2.1 mm, 1.8 μm) was used with the column temperature at 40 °C. The mobile phases were water with 0.1% formic acid (A) and acetonitrile (B). The gradient used was as follows: (0–1) min, 5%→15% B; (1–8) min, 15%→31% B; (8–16) min, 31%→35% B; (16–21) min, 35%→49% B; (21–24) min, 49%→60% B; (24–26) min, 60% B; (26–27) min, 60%→95% B; (27–28) min, 95% B; (28–29) min, 95%→5% B; and (29–31) min, 5% B. The flow rate was 0.5 mL/min. The injection volume of the sample was 1 μL. The data acquisition mode was MS^E^. Each sample was injected for ESI^−^ analyses, and data were acquired from 50 Da to 1600 Da. A QC sample containing pooled different parts was regularly injected to monitor the system stability and minimize the analytical variation.

For MS conditions: the source temperature was 110 °C, and the desolvation temperature was 550 °C, and the desolvation gas flow was 1000 L/h. The capillary voltage was 2.5 kV (ESI^−^). At a low CE scan, the cone voltage was 50 V, and the collision energy was 4 eV. At a high CE scan, the cone voltage was 50 V, and the collision energy was ramping 30–50 eV. Leucine-enkephalin was used as lock mass. The instrument was controlled by UNIFI software (version 1.9.4, Waters Corp., Milford, MA, USA).

A UHPLC-CAD analysis was performed on the Thermo Vanquish UHPLC system (ThermoFisher Scientific, Germering, Bavaria, Germany). A Phenomenex C18 column (100 × 4.6 mm, 2.6 μm) was used with a column temperature at 25 °C. The mobile phases were water with 0.1% formic acid (A) and acetonitrile (B). The gradient used was as follows: (−5–0) min, 21% B; (0–6) min, 21% B; (6–10) min, 21%→29% B; (10–21) min, 29% B; (21–26) min, 29%→35% B; (26–32) min, 35%→53% B; (32–33) min, 53%→95% B; (33–36) min, 95% B; and (36–37) min, 95%→21% B. The flow rate was 1.0 mL/min. The injection volume of the sample was 10 μL.

### 3.4. Data Analysis by the UNIFI Informatics Platform

A database of the total chemical ingredients of the ginseng (304 compounds) was created for UNIFI 1.9.4 (Waters Corporation, Milford, MA, USA) on the basis of the result of the literature and some online databases or internet search engines, such as PubMed, Full-Text Database (CNKI), SciFinder, ChemSpider, Web of Science, and Medline [19,29,38,41,42]. The database included the compound names, molecular formulas, chemical structures, and fragment ions. Data analysis was accomplished by UNIFI 1.9.4., and the parameter setting is as follows: the maximum allowed number of peak detections was 1000 for 2D peak detection, the peak intensity threshold was 25 counts for high energy and 200 counts for low energy in the 3D peak detection. The mass and fragment errors were set to be 10 mDa for chemical identification, which would be the exactly predicted fragments from the structure. We also selected +HCOO^−^, −H, and −H + 2H_2_O as adducts in ESI^−^ mode. Leucine-enkephalin was used as the reference compound to ensure the mass accuracy, and [M − H]^−^ 554.2620 was used in the negative ion.

### 3.5. Multivariate Statistical Analysis

All data acquisition in MS^E^ mode was in the continuum mode, and the raw data were processed by UNIFI 1.9.4. The data analysis included deconvolution, alignment, and data reduction to provide a list of mass and retention time pairs along with the corresponding peak areas for all detected peaks from each file in the data set. The processed data list was then imported by the PCA and OPLS-DA. All the test groups were discriminated in the PCA to investigate whether different groups could be separated. The parameters used in the analysis were 0–27 min for the retention time range, 100–1500 Da for the mass range, 0.02 Da for the mass tolerance, and 0.10 min for the retention time tolerance. The isotopic peaks were excluded for analysis. Then, OPLS-DA was carried out to discriminate ions contributing to the classification among the samples. The results were visualized in a score plot to show the group clusters and an S plot to show the variables that contribute to the classification.

## 4. Conclusions

A strategy based on UHPLC-Q-TOF/MS coupled with the UNIFI informatics platform was developed to effectively profile and characterize of ginsenosides in four parts of *P. ginseng* root. One hundred and five ginsenosides including 16 new compounds were identified or tentatively characterized. Among them, 83, 101, 99, and 96 ginsenosides were tentatively characterized in the MR, FR, BR, and RH, respectively. UHPLC-Q-TOF/MS analysis combined with multivariate statistical analysis showed an obvious difference between fibrous root and other parts. A total of 22 (20, 17, and 19 markers for MR and FR, MR and BR, and MR and RH, respectively) robust known chemical markers were identified and they were with lower contents in MR samples than other parts. The markers between MR and FR, MR and BR, and MR and RH groups included PPT-types and PPD-types. In addition, two OA-type ginsenosides (ginsenoside Ro and chikusetsusaponin Iva) were higher in RH samples than MR samples. Finally, the UHPLC-CAD semiquantitative results showed that the relative contents of Re, Rb_1_, 20(R)-Rh_1_, Rd, and Rf were highest in FR, followed by RH, BR, and MR. The relative content of Rg_1_ was highest in RH and the total content of pharmacopoeia indicators Rg_1_, Re, and Rb_1_ was highest in FR. Under our research conditions, the range of Rg_1_/Re was from 0.19 to 0.64 in FR samples, while the range of Rg_1_/Re was from 0.75 to 2.00 in MR samples. The peak area ratio of Rg_1_ and Re might be the marker of MR and FR samples.

This study systematically revealed the differences of ginsenoside components in the different parts of cultivated ginseng root. These parts were all rich in ginsenosides and contained similar structural types. The differences among them were in the compositions and relative contents of ginsenosides. Fibrous roots showed rich ingredients and high ginsenosides contents which should be further utilized. The research results provided a basis for the rational development and utilization of ginseng root.

## Figures and Tables

**Table 1:** Identification results of chemical constituents from different parts of *P. ginseng* root by UHPLC-Q-TOF/MS.

Peak No	t_R_ (min)	Formula	Observed *m/z*	Mass Error (mDa)	Adducts	Fragment Ions	Identification
1	4.42	C_42_H_72_O_15_	861.4829	−2.4	+HCOO	815.4703, 653.4206, 491.3689	Ginsenoside Re_5_ isomer [19]
2	4.58	C_54_H_92_O_24_	1169.5935	−2.6	+HCOO	1123.5872, 961.5292, 815.4739, 653.4369, 491.3802	491-(Glc-Glc)-(Rha-Glc) [19]
3	4.60	C_42_H_72_O_15_	861.4828	−3	+HCOO	815.4703, 653.4239, 491.3774	Ginsenoside Re_5_ isomer [19]
4	4.73	C_42_H_72_O_15_	861.4824	−3	+HCOO	815.4703, 653.4239, 491.3774	Ginsenoside Re_5_ isomer [19]
5	4.89	C_54_H_92_O_24_	1169.5931	−2.9	+HCOO	1123.5745, 961.5371, 799.4979, 637.4671, 475.3684	Koryoginsenoside R_2_/Ginsenoside V (24β) [19]
6	5.04	C_54_H_92_O_24_	1169.5927	−3.3	+HCOO	1123.5872, 961.5292, 829.4566, 651.4229, 489.3498	489-(Glc-GlurA)-(132-Glc) [19]
7	5.10	C_48_H_82_O_19_	1007.5396	−3.6	+HCOO	961.5331, 799.4800, 637.4253, 475.3878	Ginsenoside Re_1_/Ginsenoside Re_2_/Ginsenoside Re_3_ [7,19]
8	5.43	C_47_H_80_O_18_	977.5291	−3.6	+HCOO	931.5292, 799.4800, 637.4253, 475.3850	Ginsenoside Re_4_ [7,19]
9	5.45	C_48_H_82_O_19_	1007.5395	−3.7	+HCOO	961.5371, 799.4800, 637.4253, 475.3850	20-O-Glucosylginsenoside Rf [23]
10	5.65	C_47_H_80_O_18_	977.5289	−3.8	+HCOO	931.5176, 799.4800, 637.4414, 475.3767	Ginsenoside Re_4_ isomer [7,19]
11	5.71	C_48_H_82_O_19_	1007.5397	−3.5	+HCOO	961.5331, 799.4800, 637.4414, 475.3767	Ginsenoside Re_1_/Ginsenoside Re_2_/Ginsenoside Re_3_ [7,19]
12	5.71	C_54_H_92_O_23_	1153.5982	−2.9	+HCOO	1107.5954, 961.5331, 799.4800, 637.4414, 475.3767	Yesanchinoside E isomer [24]
13	5.73	C_53_H_90_O_22_	1123.5868	−3.8	+HCOO	1077.5829, 931.5370, 799.4800, 637.4446, 475.3767	Floralginsenoside M/Floralginsenoside N [19]
14	5.76	C_47_H_80_O_18_	977.5289	−3.8	+HCOO	931.5254, 799.4692, 637.4221, 475.3767	* Notoginsenoside R_1_
15	5.89	C_48_H_82_O_19_	1007.5396	−3.6	+HCOO	961.5331, 799.4835, 637.4318, 475.3850	Ginsenoside Re_1_/Ginsenoside Re_2_/Ginsenoside Re_3_ [7,19]
16	6.06	C_54_H_90_O_24_	1167.5773	−3.1	+HCOO	1121.5889, 959.4955, 797.5107, 635.3868, 473.3548	Notoginsenoside B [19]
17	6.07	C_54_H_92_O_23_	1153.5980	−3.1	+HCOO	1107.5869, 961.5253, 799.4764, 637.4189, 475.3740	Yesanchinoside E isomer [24]
18	6.08	C_48_H_82_O_19_	1007.5391	−4.2	+HCOO	961.5253, 799.4764, 637.4189, 475.3740	Vinaginsenoside R_4_/Notoginsenoside R_3_/Notoginsenoside R_6_/Notoginsenoside M/Notoginsenoside N isomer [19]
19	6.30	C_42_H_72_O_14_	845.4874	−3	+HCOO	799.4800, 637.4285, 475.3795	* Ginsenoside Rg_1_
20	6.31	C_48_H_82_O_18_	991.5446	−3.7	+HCOO	945.5389, 799.4800, 637.4285, 475.3795	* Ginsenoside Re
21	6.85	C_44_H_74_O_15_	887.4975	−3.4	+HCOO	841.4911, 799.4800, 637.4318, 475.3767	6′-O-Acetyl-ginsenoside Rg_1_ isomer [25]
22	6.92	C_50_H_84_O_19_	1033.5548	−4.1	+HCOO	987.5516, 945.5389, 799.4764, 637.4189, 475.3823	6′′′-O-Acetyl-ginsenoside Re isomer [19]
23	7.19	C_44_H_74_O_15_	887.4974	−3.6	+HCOO	841.4985, 799.4764, 637.4285, 475.3850	6′-O-Acetyl-ginsenoside Rg_1_ isomer [25]
24	7.23	C_50_H_84_O_19_	1033.5545	−4.4	+HCOO	987.5476, 945.5389, 799.4728, 637.4285, 475.3795	6′′′-O-Acetyl-ginsenoside Re isomer [19]
25	7.33	C_44_H_74_O_15_	887.4976	−3.4	+HCOO	841.4985, 799.4835, 637.4318, 475.3795	6′-O-Acetyl-ginsenoside Rg_1_ isomer [19]
26	7.33	C_42_H_70_O_13_	827.4774	−2.5	+HCOO	781.4672, 619.4266	Ginsenoside Rg_5_ [26]
27	7.68	C_48_H_82_O_19_	1007.5404	−2.8	+HCOO	961.5450, 799.5051, 637.4318, 475.3850	Vinaginsenoside R_4_/Notoginsenoside R_3_/Notoginsenoside R_6_/Notoginsenoside M/Notoginsenoside N isomer [19]
28	7.74	C_53_H_90_O_23_	1139.5843	−1.2	+HCOO	1093.5971, 961.5489, 799.4800, 637.4350, 475.3767	Floralginsenoside P isomer [19]
29	7.74	C_44_H_72_O_13_	869.4885	−1.9	+HCOO	823.4829, 781.4743, 619.4203	Ginsenoside Rs_4_/Ginsenoside Rs_5_ [27]
30	7.82	C_47_H_74_O_18_	971.4823	−3.4	+HCOO	925.5345, 763.4524, 631.2789, 455.2456	Pseudoginsenoside Rt_1_ [7,28]
31	7.85	C_53_H_90_O_23_	1139.5841	−1.4	+HCOO	1093.5743, 961.5341, 799.5231, 637.4318, 475.3795	Floralginsenoside P isomer [19]
32	7.96	C_44_H_74_O_15_	887.4988	−2.2	+HCOO	841.4911, 799.4943, 637.4189, 475.3740	Yesanchinoside D [7]
33	8.29	C_48_H_82_O_19_	1007.5409	−2.3	+HCOO	961.5410, 799.4728, 637.4189, 475.3712	Vinaginsenoside R_4_/Notoginsenoside R_3_/Notoginsenoside R_6_/Notoginsenoside M/Notoginsenoside N isomer [19]
34	8.31	C_42_H_72_O_15_	861.4835	−1.9	+HCOO	815.4812, 653.4271, 491.3830	Ginsenoside Re_5_ isomer [19]
35	8.39	C_42_H_70_O_14_	843.4724	−2.3	+HCOO	797.4461, 637.4382,	12,23-Eproxyginsenoside Rg_1_ [29]
36	8.42	C_48_H_82_O_19_	1007.5405	−2.7	+HCOO	961.5331, 799.4800, 637.4285	Vinaginsenoside R_4_/Notoginsenoside R_3_/Notoginsenoside R_6_/Notoginsenoside M/Notoginsenoside N isomer [19]
37	8.84	C_48_H_82_O_19_	1007.5403	−2.9	+HCOO	961.5371, 799.4835, 637.4285, 475.3767	Vinaginsenoside R_4_/Notoginsenoside R_3_/Notoginsenoside R_6_/Notoginsenoside M/Notoginsenoside N isomer [19]
38	9.12	C_54_H_86_O_24_	1117.5409	−2.7	−H	1117.5296, 945.4959, 869.4686, 783.9150, 621.4222, 459.3493	malnoylfloralginsenosides Rd6/β-D-Glucopyranoside, (3β, 12β)-20-(β-D-glucopyranosyloxy)-12-hydroxydammar-24-en-3-yl 2-O-[6-O-(2-carboxyacetyl)-β-D-glucopyranosyl]-, 6-(hydrogen propanedioate) [30]
39	9.15	C_42_H_72_O_14_	845.4881	−2.3	+HCOO	799.4835, 637.4318, 475.3823	* Ginsenoside Rf
40	9.38	C_46_H_76_O_15_	913.5142	−2.4	+HCOO	867.4936, 799.4871, 637.4350, 475.3823	Ginsenoside Re_6_/Koryoginsenoside R_1_ [7,19,31]
41	9.75	C_44_H_74_O_15_	887.4980	−3	+HCOO	841.4948, 799.4871, 637.4318, 475.3795	Yesanchinoside D isomer [7]
42	9.77	C_59_H_100_O_27_	1285.6409	−2.5	+HCOO	1239.6308, 1107.5700, 1077.6080, 945.5623, 783.4913, 621.4317, 459.3929	Notoginsenoside R_4_ [20]
43	9.81	C_41_H_70_O_13_	815.4777	−2.2	+HCOO	769.4657, 637.4318, 475.3795	* Notoginsenoside R_2_
44	10.14	C_61_H_102_O_28_	1327.6498	−4.1	+HCOO	1281.6390, 1239.6218, 1149.5938, 1107.6123, 1077.5704, 945.5350, 783.4949, 621.4349, 459.3820	Yesanchinoside J isomer [32]
45	10.14	C_62_H_102_O_30_	1325.636	−2.3	−H	1325.6340, 1239.6218, 1107.6123, 1077.5704, 945.5350, 783.4949, 621.4349, 459.3820	malonyl-ginsenoside Ra_3_/malonyl-notoginsenoside R_4_ [10,33]
46	10.58	C_42_H_72_O_13_	829.4936	−1.9	+HCOO	783.4878, 637.4350, 475.3795	* Ginsenoside Rg_2_
47	10.75	C_36_H_62_O_9_	683.4370	−0.6	+HCOO	637.4319, 475.3767	* 20(R)-Ginsenoside Rh_1_
48	11.19	C_58_H_98_O_26_	1255.6295	−3.3	+HCOO	1209.6187, 1077.5704, 1047.5752, 945.5389, 915.5220, 783.4842, 621.4317, 459.3847	Ginsenoside Ra_2_ [34]
49	11.39	C_59_H_100_O_27_	1285.6403	−3.1	+HCOO	1239.6263, 1107.5954, 1077.5913, 945.5311, 915.5143, 783.4913, 621.4285, 459.3847	Ginsenoside Ra_3_ [34]
50	11.49	C_54_H_92_O_23_	1153.5979	−3.2	+HCOO	1107.5911, 945.5271, 783.4878, 621.4349, 459.3874	* Ginsenoside Rb_1_
51	11.66	C_60_H_100_O_27_	1297.6387	−4.7	+HCOO	1251.6324, 1209.6231, 1077.6038, 1047.5340, 945.5389, 915.5028, 783.4842, 621.4380, 459.3847	Ginsenoside Ra_5_/(3β, 12β)-3-[[2-O-(6-O-Acetyl-β-D-glucopyranosyl)-β-D-glucopyranosyl]oxy]-12-hydroxydammar-24-en-20-yl O-β-D-xylopyranosyl-(1→2)-O-α-L-arabinopyranosyl-(1→6)-β-D-glucopyranoside isomer [9]
52	11.87	C_61_H_102_O_28_	1327.6486	−5.4	+HCOO	1281.6435, 1239.6263, 1149.6110, 1107.5911, 1077.5871, 945.5389, 783.4842, 621.4390, 459.3902	Yesanchinoside J isomer [32]
53	11.93	C_48_H_76_O_19_	955.4868	−4	−H	955.4857, 793.4326, 631.4035, 455.3596	* Ginsenoside Ro
54	11.99	C_56_H_94_O_24_	1195.6074	−4.4	+HCOO	1149.5981, 1107.5996, 987.5396, 945.5428, 783.4878, 621.4285, 459.3820	Quinquenoside R_1_/(3β, 12β)-20-[[6-O-(6-O-Acetyl-β-D-glucopyranosyl)-β-D-glucopyranosyl]oxy]-12-hydroxydammar-24-en-3-yl 2-O-β-D-glucopyranosyl-β-D-glucopyranoside isomer [26]
55	12.00	C_57_H_94_O_26_	1193.5924	−3.6	−H	1193.5948, 1107.5996, 945.5389, 783.4771, 621.4285, 459.3738	malonyl-ginsenoside Rb_1_ [26]
56	12.42	C_53_H_90_O_22_	1123.5869	−3.7	+HCOO	1077.5787, 945.5350, 915.5143, 783.4842, 621.4349, 459.3820	* Ginsenoside Rc
57	12.47	C_58_H_98_O_26_	1255.6298	−3.1	+HCOO	1209.6231, 1077.5787, 1047.5669, 945.5506, 915.5374, 783.4771, 621.4539, 459.3820	Ginsenoside Ra_1_ [26]
58	12.92	C_60_H_100_O_27_	1297.6383	−5.1	+HCOO	1251.6324, 1209.6187, 1077.5746, 1047.5793, 945.5389, 915.5259, 783.4878, 621.4349, 459.3874	Ginsenoside Ra_5_/(3β, 12β)-3-[[2-O-(6-O-Acetyl-β-D-glucopyranosyl)-β-D-glucopyranosyl]oxy]-12-hydroxydammar-24-en-20-yl O-β-D-xylopyranosyl-(1→2)-O-α-L-arabinopyranosyl-(1→6)-β-D-glucopyranoside isomer [9]
59	13.00	C_55_H_92_O_23_	1165.5969	−4.3	+HCOO	1119.5924, 1077.5871, 987.5676, 945.5506, 915.5297, 783.4878, 621.4475, 459.3820	Ginsenoside Rs_1_/Ginsenoside Rs_2_/Pseudoginsenoside F_8_ isomer [7,35]
60	13.01	C_56_H_94_O_25_	1163.5818	−3.7	−H	1163.5717, 1077.5871, 945.5506, 915.5297, 783.4878, 621.4475, 459.3820	malonyl-ginsenoside Rc [26]
61	13.32	C_56_H_94_O_24_	1195.6077	−4.1	+HCOO	1149.6003, 1107.5871, 987.5665, 945.5438, 783.4823, 621.4246, 459.3792	Quinquenoside R_1_/(3β, 12β)-20-[[6-O-(6-O-Acetyl-β-D-glucopyranosyl)-β-D-glucopyranosyl]oxy]-12-hydroxydammar-24-en-3-yl 2-O-β-D-glucopyranosyl-β-D-glucopyranoside isomer [26]
62	13.34	C_58_H_98_O_26_	1255.6297	−3.1	+HCOO	1209.6149, 1077.5859, 1047.6303, 945.5438, 915.5641, 783.4823, 621.4246, 459.3792	Ginsenoside Ra_1_ isomer [26]
63	13.43	C_53_H_90_O_22_	1123.5865	−4	+HCOO	1077.5787, 945.5350, 915.5259, 783.4806, 621.4349, 459.3847	Ginsenoside Rb_2_ [9]
64	13.76	C_56_H_94_O_24_	1195.6075	−4.2	+HCOO	1149.6110, 1107.5996, 945.5389, 783.4913, 621.4317, 459.3793	Quinquenoside R_1_/(3β, 12β)-20-[[6-O-(6-O-Acetyl-β-D-glucopyranosyl)-β-D-glucopyranosyl]oxy]-12-hydroxydammar-24-en-3-yl 2-O-β-D-glucopyranosyl-β-D-glucopyranoside isomer [26]
65	13.78	C_53_H_90_O_22_	1123.5871	−3.5	+HCOO	1077.5787, 945.5350, 915.5182, 783.4842, 621.4317, 459.3956	Ginsenoside Rb_3_ [9]
66	13.89	C_60_H_100_O_27_	1297.6400	−3.4	+HCOO	1251.6369, 1209.6143, 1077.5620, 945.5076, 915.4990, 783.5056, 621.4412, 459.3874	Ginsenoside Ra_5_/(3β, 12β)-3-[[2-O-(6-O-Acetyl-β-D-glucopyranosyl)-β-D-glucopyranosyl]oxy]-12-hydroxydammar-24-en-20-yl O-β-D-xylopyranosyl-(1→2)-O-α-L-arabinopyranosyl-(1→6)-β-D-glucopyranoside isomer [9]
67	14.00	C_55_H_92_O_23_	1165.5970	−4.2	+HCOO	1119.5881, 1077.5746, 987.5396, 945.5350, 915.5297, 783.4878, 621.4349, 459.3738	Ginsenoside Rs_1_/Ginsenoside Rs_2_/Pseudoginsenoside F_8_ isomer [7,35]
68	14.01	C_56_H_94_O_25_	1163.5819	−3.6	−H	1163.5934, 1077.5746, 945.5350, 915.5297, 783.4878, 621.4349, 459.3738	malonyl-ginsenoside Rb_2_ [26]
69	14.36	C_55_H_92_O_23_	1165.5972	−4	+HCOO	1119.5924, 1077.5746, 987.5596, 945.5300, 915.5297, 783.4806, 621.4349, 459.3902	Ginsenoside Rs_1_/Ginsenoside Rs_2_/Pseudoginsenoside F_8_ isomer [7,35]
70	14.42	C_60_H_100_O_27_	1297.6398	−3.6	+HCOO	1251.6279, 1209.6231, 1077.5787, 945.5467, 915.5297, 783.4771, 621.4317, 459.3793	Ginsenoside Ra_5_/(3β, 12β)-3-[[2-O-(6-O-Acetyl-β-D-glucopyranosyl)-β-D-glucopyranosyl]oxy]-12-hydroxydammar-24-en-20-yl O-β-D-xylopyranosyl-(1→2)-O-α-L-arabinopyranosyl-(1→6)-β-D-glucopyranoside isomer [9]
71	14.44	C_55_H_92_O_23_	1165.5971	−4	+HCOO	1119.5924, 1077.5787, 987.5396, 945.5467, 915.5297, 783.4771, 621.4317, 459.3793	Ginsenoside Rs_1_/Ginsenoside Rs_2_/Pseudoginsenoside F_8_ isomer [7,35]
72	14.69	C_60_H_100_O_27_	1297.6412	−2.2	+HCOO	1251.6279, 1209.6231, 1077.5787, 945.5467, 915.5143, 783.4913, 621.4222, 459.3793	Ginsenoside Ra_5_/(3β, 12β)-3-[[2-O-(6-O-Acetyl-β-D-glucopyranosyl)-β-D-glucopyranosyl]oxy]-12-hydroxydammar-24-en-20-yl O-β-D-xylopyranosyl-(1→2)-O-α-L-arabinopyranosyl-(1→6)-β-D-glucopyranoside isomer [9]
73	14.88	C_60_H_100_O_27_	1297.6412	−2.2	+HCOO	1251.6279, 1209.6231, 1077.5787, 945.5467, 915.5143, 783.4913, 621.4222, 459.3793	Ginsenoside Ra_5_/(3β, 12β)-3-[[2-O-(6-O-Acetyl-β-D-glucopyranosyl)-β-D-glucopyranosyl]oxy]-12-hydroxydammar-24-en-20-yl O-β-D-xylopyranosyl-(1→2)-O-α-L-arabinopyranosyl-(1→6)-β-D-glucopyranoside isomer [9]
74	14.89	C_56_H_94_O_24_	1195.6097	−2	+HCOO	1149.6024, 1107.5700, 945.5467, 783.4913, 621.4222, 459.3793	Quinquenoside R_1_/(3β, 12β)-20-[[6-O-(6-O-Acetyl-β-D-glucopyranosyl)-β-D-glucopyranosyl]oxy]-12-hydroxydammar-24-en-3-yl 2-O-β-D-glucopyranosyl-β-D-glucopyranoside isomer [26]
75	14.93	C_42_H_66_O_14_	793.4361	−1.8	−H	793.4362, 631.3811, 455.3623	Chikusetsusaponin Iva [26]
76	14.96	C_55_H_92_O_23_	1165.5985	−2.7	+HCOO	1119.5966, 1077.5787, 945.5428, 915.5220, 783.4984, 621.4380, 459.3874	Ginsenoside Rs_1_/Ginsenoside Rs_2_/Pseudoginsenoside F_8_ isomer [7,35]
77	15.60	C_55_H_92_O_23_	1165.5974	−3.7	+HCOO	1119.5881, 1077.5787, 945.5350, 915.522, 783.4735, 621.438, 459.3874	Ginsenoside Rs_1_/Ginsenoside Rs_2_/Pseudoginsenoside F_8_ isomer [7,35]
78	15.61	C_56_H_94_O_25_	1163.5826	−2.9	−H	1163.5543, 1077.5787, 945.5350, 915.522, 783.4735, 621.438, 459.3874	malonyl-ginsenoside Rb_3_ [26]
79	15.82	C_48_H_82_O_18_	991.5443	−4.1	+HCOO	945.5389, 783.4842, 621.4317, 459.3847	* Ginsenoside Rd
80	16.50	C_50_H_84_O_19_	1033.5548	−4.1	+HCOO	987.5516, 945.5389, 825.4985, 783.4878, 621.4412, 459.3847	Pseudoginsenoside Rc_1_/Quinquenoside III/β-D-Glucopyranoside, (3β, 12β)-3-(β-D-glucopyranosyloxy)-12-hydroxydammar-24-en-20-yl 6-O-(6-O-acetyl-β-D-glucopyranosyl)-(9CI) isomer [36]
81	16.92	C_50_H_84_O_19_	1033.5549	−4	+HCOO	987.5436, 945.5430, 825.4912, 783.4878, 621.4349, 459.3820	Pseudoginsenoside Rc_1_/Quinquenoside III/β-D-Glucopyranoside, (3β, 12β)-3-(β-D-glucopyranosyloxy)-12-hydroxydammar-24-en-20-yl 6-O-(6-O-acetyl-β-D-glucopyranosyl)-(9CI) isomer [36]
82	17.34	C_55_H_92_O_23_	1165.5987	−2.5	+HCOO	1119.5924, 1077.5746, 945.5663, 915.5297, 783.6764, 621.4507, 459.1830	Ginsenoside Rs_1_/Ginsenoside Rs_2_/Pseudoginsenoside F_8_ isomer [7,35]
83	17.44	C_53_H_88_O_22_	1075.5656	−3.9	+HCOO	1029.5511, 987.5436, 945.5389, 783.4842, 621.4349, 459.3847	459-Glc-Glc-Glc-2Acetyl
84	17.44	C_54_H_86_O_24_	1117.5403	−3.4	−H	1117.4956, 1031.5678, 945.5389, 783.4842, 621.4349, 459.3847	malnoylfloralginsenosides Rd6/β-D-Glucopyranoside, (3β, 12β)-20-(β-D-glucopyranosyloxy)-12-hydroxydammar-24-en-3-yl 2-O-[6-O-(2-carboxyacetyl)-β-D-glucopyranosyl]-, 6-(hydrogen propanedioate) [30]
85	17.83	C_48_H_82_O_18_	991.5450	−3.3	+HCOO	945.5389, 783.4777, 621.4285, 459.3820	Gypenoside XVII [20]
86	17.84	C_50_H_84_O_19_	1033.5550	−3.9	+HCOO	987.5436, 945.5389, 825.4985, 783.4735, 621.4285, 459.3820	Pseudoginsenoside Rc_1_/Quinquenoside III/β-D-Glucopyranoside, (3β, 12β)-3-(β-D-glucopyranosyloxy)-12-hydroxydammar-24-en-20-yl 6-O-(6-O-acetyl-β-D-glucopyranosyl)-(9CI) isomer [36]
87	17.93	C_58_H_96_O_24_	1221.6242	−3.2	+HCOO	1175.6144, 1107.5954, 945.5311, 783.4735, 621.4190, 459.3874	Ginsenoside Ra_6_/Quinquenoside II [37]
88	18.06	C_51_H_84_O_21_	1031.5422	−1	−H	1031.5597, 945.5428, 783.4842, 621.4697, 459.3166	malonyl-ginsenoside Rd [26]
89	18.15	C_50_H_84_O_19_	1033.5550	−3.9	+HCOO	987.5516, 945.5311, 825.4766, 783.4842, 621.4380, 459.3929	Pseudoginsenoside Rc_1_/Quinquenoside III/β-D-Glucopyranoside, (3β, 12β)-3-(β-D-glucopyranosyloxy)-12-hydroxydammar-24-en-20-yl 6-O-(6-O-acetyl-β-D-glucopyranosyl)-(9CI) or isomer [36]
90	18.33	C_53_H_88_O_22_	1075.5661	−3.4	−H	1029.5633, 987.5476, 945.5389, 783.4842, 621.4412, 459.3138	459-Glc-Glc-Glc-2Acetyl isomer
91	18.64	C_57_H_94_O_23_	1191.6136	−3.2	+HCOO	1145.6045, 1077.5704, 945.5193, 915.5182, 783.4842, 621.4349, 459.3983	Ginsenoside Ra_7_ [37]
92	18.73	C_47_H_80_O_17_	961.5347	−3	+HCOO	915.5413, 783.4913, 753.4709, 621.4381, 459.3738	Gypenoside IX [26]
93	18.90	C_50_H_84_O_19_	1033.5555	−3.4	+HCOO	987.5396, 945.5271, 825.4803, 783.4806, 621.4349, 459.3929	Pseudoginsenoside Rc_1_/Quinquenoside III/β-D-Glucopyranoside, (3β, 12β)-3-(β-D-glucopyranosyloxy)-12-hydroxydammar-24-en-20-yl 6-O-(6-O-acetyl-β-D-glucopyranosyl)-(9CI) isomer [36]
94	19.14	C_57_H_94_O_23_	1191.6134	−3.4	+HCOO	1145.6002, 1077.5787, 945.5545, 915.5182, 783.4842, 621.4412, 459.3111	Ginsenoside Ra_8_/Ginsenoside Ra_9_ [37]
95	20.23	C_42_H_66_O_14_	793.4360	−2	−H	793.4326, 631.3907, 455.3514	Chikusetsusaponin Iva isomer [26]
96	20.34	C_52_H_86_O_19_	1059.5710	−3.6	+HCOO	1013.5490, 945.5467, 783.4949, 621.4444, 459.2893	Quinquenoside I [36]
97	20.58	C_42_H_72_O_13_	829.4928	−2.7	+HCOO	783.4949, 621.4349, 459.3738	* (20S)-Ginsenoside Rg_3_
98	20.60	C_57_H_94_O_23_	1191.6162	−0.6	+HCOO	1145.6519, 1077.5662, 945.5545, 915.5451, 783.4949, 621.4254, 459.3738	Ginsenoside Ra_8_/Ginsenoside Ra_9_ [37]
99	20.87	C_44_H_74_O_14_	871.5036	−2.5	+HCOO	825.5036, 783.4842, 621.4444, 459.3683	20(S)-Ginsenoside Rs_3_ [27]
100	21.44	C_42_H_72_O_13_	829.4936	−1.9	+HCOO	783.4949, 621.4380, 459.3765	* (20R)-Ginsenoside Rg_3_
101	21.57	C_44_H_74_O_14_	871.5036	−2.4	+HCOO	825.5058, 783.4842, 621.4380, 459.3820	(20R)-Ginsenoside Rs_3_ [8]
102	24.25	C_42_H_70_O_12_	811.4847	−0.2	+HCOO	765.4741, 603.4275, 441.1546	Ginsenoside Rk_1_/Ginsenoside Rz_1_ [38,39]
103	24.53	C_36_H_62_O_8_	667.4426	−0.1	+HCOO	621.4317, 459.4011	* 20(S)-Ginsenoside Rh_2_
104	24.56	C_36_H_62_O_8_	667.4426	−0.1	+HCOO	621.3524, 459.4038	* 20(R)-Ginsenoside Rh_2_
105	24.62	C_42_H_70_O_12_	811.4847	−0.2	+HCOO	765.4530, 603.3338, 441.2454	Ginsenoside Rk_1_/Ginsenoside Rz_1_ [38,39]

* Compared with reference standards.

**Table 2:** The screened marker compounds for the discrimination of the different parts of *P. ginseng* root.

Source	Peak No	t_R_ (min)	Observed *m*/*z*	Adducts	Aglycone Type	Identification	Main Existing Groups
MR and FR	20	6.31	991.5446	+HCOO	PPT	Ginsenoside Re	Fibrous root
	24	7.23	1033.5545	+HCOO	PPT	6′′′-O-Acetyl-ginsenoside Re/isomer	Fibrous root
	39	9.15	845.4881	+HCOO	PPT	Ginsenoside Rf	Fibrous root
	46	10.58	829.4936	+HCOO	PPT	Ginsenoside Rg_2_	Fibrous root
	48	11.19	1255.6295	+HCOO	PPD	Ginsenoside Ra_2_	Fibrous root
	50	11.49	1153.5979	+HCOO	PPD	Ginsenoside Rb_1_	Fibrous root
	51	11.66	1297.6387	+HCOO	PPD	Ginsenoside Ra_5_/(3β, 12β)-3-[[2-O-(6-O-Acetyl-β-D-glucopyranosyl)-β-D-glucopyranosyl]oxy]-12-hydroxydammar-24-en-20-yl O-β-D-xylopyranosyl-(1→2)-O-α-L-arabinopyranosyl-(1→6)-β-D-glucopyranoside or isomer	Fibrous root
	54	11.99	1195.6074	+HCOO	PPD	Quinquenoside R_1_/(3β, 12β)-20-[[6-O-(6-O-Acetyl-β-D-glucopyranosyl)-β-D-glucopyranosyl]oxy]-12-hydroxydammar-24-en-3-yl 2-O-β-D-glucopyranosyl-β-D-glucopyranoside or isomer	Fibrous root
	56	12.42	1123.5869	+HCOO	PPD	Ginsenoside Rc	Fibrous root
	59	13.00	1165.5969	+HCOO	PPD	Ginsenoside Rs_1_/Ginsenoside Rs_2_/Pseudoginsenoside F_8_ isomer	Fibrous root
	61	13.32	1195.6077	+HCOO	PPD	Quinquenoside R_1_/(3β, 12β)-20-[[6-O-(6-O-Acetyl-β-D-glucopyranosyl)-β-D-glucopyranosyl]oxy]-12-hydroxydammar-24-en-3-yl 2-O-β-D-glucopyranosyl-β-D-glucopyranoside or isomer	Fibrous root
	63	13.43	1123.5865	+HCOO	PPD	Ginsenoside Rb_2_	Fibrous root
	65	13.78	1123.5871	+HCOO	PPD	Ginsenoside Rb_3_	Fibrous root
	67	14.00	1165.597	+HCOO	PPD	Ginsenoside Rs_1_/Ginsenoside Rs_2_/Pseudoginsenoside F_8_ isomer	Fibrous root
	69	14.36	1165.5972	+HCOO	PPD	Ginsenoside Rs_1_/Ginsenoside Rs_2_/Pseudoginsenoside F_8_ isomer	Fibrous root
	77	15.60	1165.5974	+HCOO	PPD	Ginsenoside Rs_1_/Ginsenoside Rs_2_/Pseudoginsenoside F_8_ isomer	Fibrous root
	79	15.82	991.5443	+HCOO	PPD	Ginsenoside Rd	Fibrous root
	80	16.50	1033.5548	+HCOO	PPD	Pseudoginsenoside Rc_1_/Quinquenoside III/β-D-Glucopyranoside, (3β, 12β)-3-(β-D-glucopyranosyloxy)-12-hydroxydammar-24-en-20-yl 6-O-(6-O-acetyl-β-D-glucopyranosyl)-(9CI) or isomer	Fibrous root
	81	16.92	1033.5549	+HCOO	PPD	Pseudoginsenoside Rc_1_/Quinquenoside III/β-D-Glucopyranoside, (3β, 12β)-3-(β-D-glucopyranosyloxy)-12-hydroxydammar-24-en-20-yl 6-O-(6-O-acetyl-β-D-glucopyranosyl)-(9CI) or isomer	Fibrous root
	86	17.84	1033.555	+HCOO	PPD	Pseudoginsenoside Rc_1_/Quinquenoside III/β-D-Glucopyranoside, (3β, 12β)-3-(β-D-glucopyranosyloxy)-12-hydroxydammar-24-en-20-yl 6-O-(6-O-acetyl-β-D-glucopyranosyl)-(9CI) or isomer	Fibrous root
MR and BR	20	6.31	991.5446	+HCOO	PPT	Ginsenoside Re	Branch root
	24	7.23	1033.5545	+HCOO	PPT	6′′′-O-Acetyl-ginsenoside Re/isomer	Branch root
	39	9.15	845.4881	+HCOO	PPT	Ginsenoside Rf	Branch root
	46	10.58	829.4936	+HCOO	PPT	Ginsenoside Rg_2_	Branch root
	50	11.49	1153.5979	+HCOO	PPD	Ginsenoside Rb_1_	Branch root
	54	11.99	1195.6074	+HCOO	PPD	Quinquenoside R_1_/(3β, 12β)-20-[[6-O-(6-O-Acetyl-β-D-glucopyranosyl)-β-D-glucopyranosyl]oxy]-12-hydroxydammar-24-en-3-yl 2-O-β-D-glucopyranosyl-β-D-glucopyranoside or isomer	Branch root
	56	12.42	1123.5869	+HCOO	PPD	Ginsenoside Rc	Branch root
	59	13.00	1165.5969	+HCOO	PPD	Ginsenoside Rs_1_/Ginsenoside Rs_2_/Pseudoginsenoside F_8_ isomer	Branch root
	61	13.32	1195.6077	+HCOO	PPD	Quinquenoside R_1_/(3β, 12β)-20-[[6-O-(6-O-Acetyl-β-D-glucopyranosyl)-β-D-glucopyranosyl]oxy]-12-hydroxydammar-24-en-3-yl 2-O-β-D-glucopyranosyl-β-D-glucopyranoside or isomer	Branch root
	63	13.43	1123.5865	+HCOO	PPD	Ginsenoside Rb_2_	Branch root
	67	14.00	1165.597	+HCOO	PPD	Ginsenoside Rs_1_/Ginsenoside Rs_2_/Pseudoginsenoside F_8_ isomer	Branch root
	69	14.36	1165.5972	+HCOO	PPD	Ginsenoside Rs_1_/Ginsenoside Rs_2_/Pseudoginsenoside F_8_ isomer	Branch root
	77	15.60	1165.5974	+HCOO	PPD	Ginsenoside Rs_1_/Ginsenoside Rs_2_/Pseudoginsenoside F_8_ isomer	Branch root
	79	15.82	991.5443	+HCOO	PPD	Ginsenoside Rd	Branch root
	80	16.50	1033.5548	+HCOO	PPD	Pseudoginsenoside Rc_1_/Quinquenoside III/β-D-Glucopyranoside, (3β, 12β)-3-(β-D-glucopyranosyloxy)-12-hydroxydammar-24-en-20-yl 6-O-(6-O-acetyl-β-D-glucopyranosyl)-(9CI) or isomer	Branch root
	81	16.92	1033.5549	+HCOO	PPD	Pseudoginsenoside Rc_1_/Quinquenoside III/β-D-Glucopyranoside, (3β, 12β)-3-(β-D-glucopyranosyloxy)-12-hydroxydammar-24-en-20-yl 6-O-(6-O-acetyl-β-D-glucopyranosyl)-(9CI) or isomer	Branch root
	86	17.84	1033.555	+HCOO	PPD	Pseudoginsenoside Rc_1_/Quinquenoside III/β-D-Glucopyranoside, (3β, 12β)-3-(β-D-glucopyranosyloxy)-12-hydroxydammar-24-en-20-yl 6-O-(6-O-acetyl-β-D-glucopyranosyl)-(9CI) or isomer	Branch root
MR and RH	20	6.31	991.5446	+HCOO	PPT	Ginsenoside Re	Rhizome
	24	7.23	1033.5545	+HCOO	PPT	6′′′-O-Acetyl-ginsenoside Re/isomer	Rhizome
	39	9.15	845.4881	+HCOO	PPT	Ginsenoside Rf	Rhizome
	46	10.58	829.4936	+HCOO	PPT	Ginsenoside Rg_2_	Rhizome
	50	11.49	1153.5979	+HCOO	PPD	Ginsenoside Rb_1_	Rhizome
	53	11.93	955.4868	+HCOO	OA	Ginsenoside Ro	Rhizome
	54	11.99	1195.6074	+HCOO	PPD	Quinquenoside R_1_/(3β, 12β)-20-[[6-O-(6-O-Acetyl-β-D-glucopyranosyl)-β-D-glucopyranosyl]oxy]-12-hydroxydammar-24-en-3-yl 2-O-β-D-glucopyranosyl-β-D-glucopyranoside or isomer	Rhizome
	56	12.42	1123.5869	+HCOO	PPD	Ginsenoside Rc	Rhizome
	59	13.00	1165.5969	+HCOO	PPD	Ginsenoside Rs_1_/Ginsenoside Rs_2_/Pseudoginsenoside F_8_ isomer	Rhizome
	61	13.32	1195.6077	+HCOO	PPD	Quinquenoside R_1_/(3β, 12β)-20-[[6-O-(6-O-Acetyl-β-D-glucopyranosyl)-β-D-glucopyranosyl]oxy]-12-hydroxydammar-24-en-3-yl 2-O-β-D-glucopyranosyl-β-D-glucopyranoside or isomer	Rhizome
	63	13.43	1123.5865	+HCOO	PPD	Ginsenoside Rb_2_	Rhizome
	67	14.00	1165.597	+HCOO	PPD	Ginsenoside Rs_1_/Ginsenoside Rs_2_/Pseudoginsenoside F_8_ isomer	Rhizome
	69	14.36	1165.5972	+HCOO	PPD	Ginsenoside Rs_1_/Ginsenoside Rs_2_/Pseudoginsenoside F_8_ isomer	Rhizome
	75	14.93	793.4361	−H	OA	Chikusetsusaponin Iva	Rhizome
	77	15.60	1165.5974	+HCOO	PPD	Ginsenoside Rs_1_/Ginsenoside Rs_2_/Pseudoginsenoside F_8_ isomer	Rhizome
	79	15.82	991.5443	+HCOO	PPD	Ginsenoside Rd	Rhizome
	80	16.50	1033.5548	+HCOO	PPD	Pseudoginsenoside Rc_1_/Quinquenoside III/β-D-Glucopyranoside, (3β, 12β)-3-(β-D-glucopyranosyloxy)-12-hydroxydammar-24-en-20-yl 6-O-(6-O-acetyl-β-D-glucopyranosyl)-(9CI) or isomer	Rhizome
	81	16.92	1033.5549	+HCOO	PPD	Pseudoginsenoside Rc_1_/Quinquenoside III/β-D-Glucopyranoside, (3β, 12β)-3-(β-D-glucopyranosyloxy)-12-hydroxydammar-24-en-20-yl 6-O-(6-O-acetyl-β-D-glucopyranosyl)-(9CI) or isomer	Rhizome
	86	17.84	1033.5550	+HCOO	PPD	Pseudoginsenoside Rc_1_/Quinquenoside III/β-D-Glucopyranoside, (3β, 12β)-3-(β-D-glucopyranosyloxy)-12-hydroxydammar-24-en-20-yl 6-O-(6-oot niteO-acetyl-β-D-glucopyranosyl)-(9CI) or isomer	Rhizome

Peak numbers were consistent with Table 1.

## Data Availability

The data presented in this study are available on request from the corresponding author. The data are not publicly available due to the protection for the former research of this study.

## Supplementary Materials

The following are available online, Figure S1: High collision energy ESI-MS spectra of peaks 51(A), 54(B), 59(C), 75(D), 80(E), and 83(F), Table S1: Chemical structures of the detected compounds in different parts of P. ginseng root, Table S2: Detailed information of the tested P. ginseng whole root samples.

## Sample Availability

Samples of the compounds are not available from the authors.
