# Supplementary material for: Comprehensive Investigation on Ginsenosides in Different Parts of a Garden-Cultivated Ginseng Root and Rhizome
Source: Molecules. 2021 Mar 18;26(6):1696. doi: 10.3390/molecules26061696 (PMC8003075; doi:10.3390/molecules26061696)
Supplement: Supplementary file 1 [file molecules-26-01696-s001.pdf]

## Supplementary data

Figure S1. High collision energy ESI-MS spectra of peaks 51(A), 54(B), 59(C), 75(D), 80(E), and 83(F).

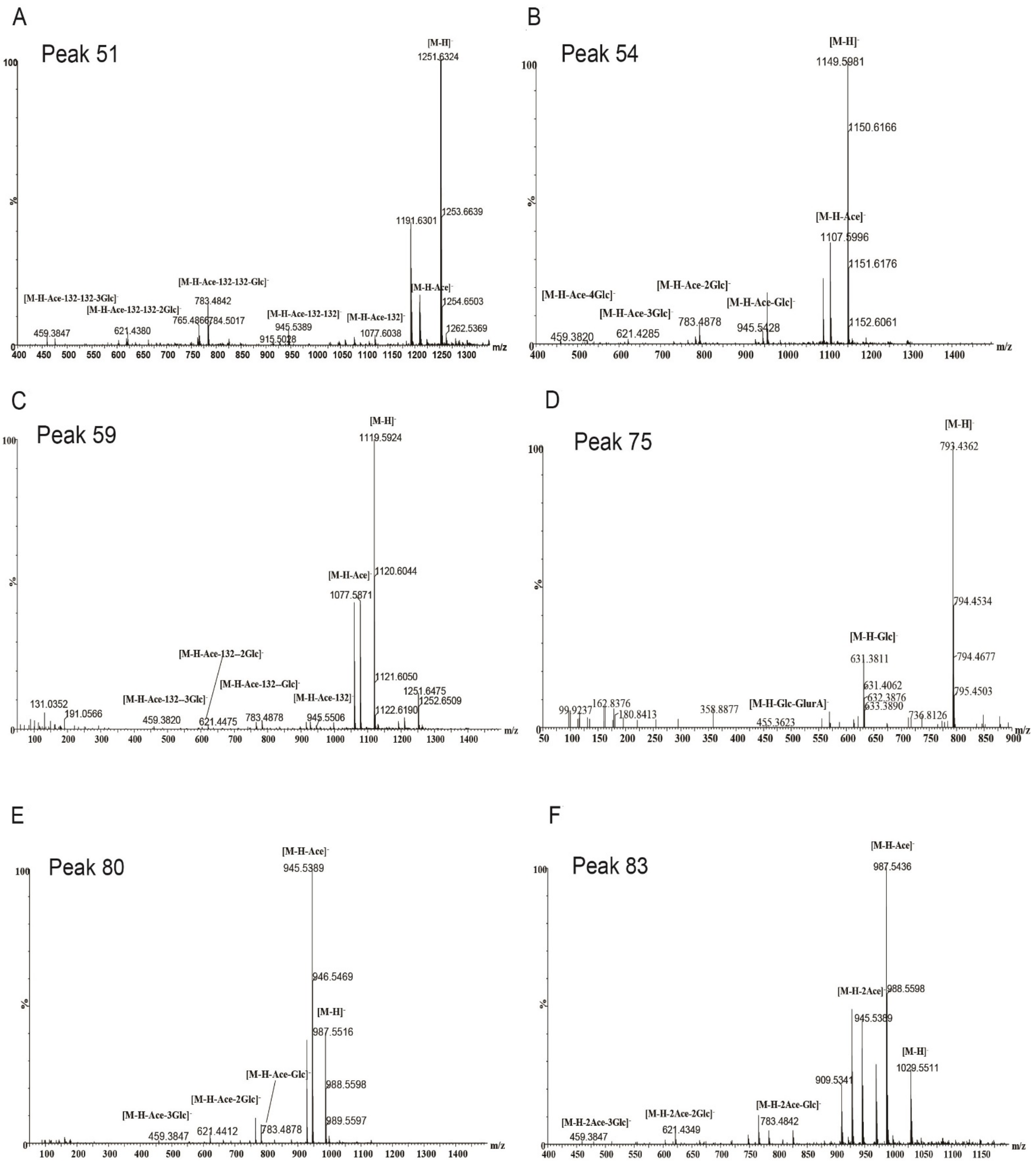

Table S1-1. Chemical structures of the detected compounds in different parts of *P*. *ginseng* root (PPD-type).

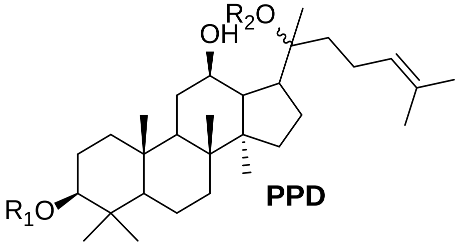


| No | Name | R1 | R2 |
|---|---|---|---|
| 38/84 | malnoylfloralginsenosides Rd$_6$ | Glc(2,1)Glc(6)-Mal | Glc(6)-Mal |
| 38/84 | β-D-Glucopyranoside, (3β,12β)-20-(β-D-glucopyranosyloxy)-12-hydroxydammar-24-en-3-yl 2-O-[6-O-(2-carboxyacetyl)-β-D-glucopyranosyl]-, 6-(hydrogen propanedioate) | Glc-[6-Mal]-(2,1)Glc-[6-Mal] | Glc |
| 42 | Notoginsenoside R$_4$ | Glc(2,1)Glc | Glc(6,1)Glc(3,1)Xyl |
| 44/52 | Yesanchinoside J | Glc-[6-Ace]-(2,1)Glc | Glc(6,1)Glc(6,1)Xyl |
| 45 | malonyl-ginsenoside Ra$_3$/ malonyl-notoginsenoside R$_4$ | Glc(2,1)Glc(6)-Mal | Glc(6,1)Glc(3,1)Xyl/Glc(6,1)Glc(6,1)Xyl |
| 48 | Ginsenoside Ra$_2$ | Glc(2,1)Glc | Glc(6,1)Ara(*f* 2,1) Xyl |
| 49 | Ginsenoside Ra$_3$ | Glc(2,1)Glc | Glc(6,1)Glc(3,1)Xyl |
| 50 | Rb$_1$ | Glc(2,1)Glc | Glc(6,1)Glc |
| 51/58/66/70/72/73 | Ra$_5$ | Glc(2,1)Glc(6)-Ace | Glc(6,1)Ara(*p* 4,1)Xyl |

| | | | |
|---|---|---|---|
| 51/58/66/70/72/73 | (3β,12β)-3-[[2-O-(6-O-Acetyl-β-D-glucopyranosyl)-β-D-glucopyranosyl]oxy]-12-hydroxydammar-24-en-20-yl O-β-D-xylopyranosyl-(1→2)-O-α-L-arabinopyranosyl-(1→6)-β-D-glucopyranoside | Glc(2,1)Glc(6)-Ace | Glc(6,1)Ara(*p* 2,1)Xyl |
| 56 | Rc | Glc(2,1)Glc | Glc(6,1)Ara(*f*) |
| 57/62 | $Ra_1$ | Glc(2,1)Glc | Glc(6,1)Ara(*p* 4,1)Xyl |
| 54/61/64/74 | Quinquenoside $R_1$ | Glc(2,1)Glc(6)-Ace | Glc(6,1)Glc |
| 54/61/64/74 | (3β,12β)-20-[[6-O-(6-O-Acetyl-β-D-glucopyranosyl)-β-D-glucopyranosyl]oxy]-12-hydroxydammar-24-en-3-yl 2-O-β-D-glucopyranosyl-β-D-glucopyranoside | Glc(2,1)Glc | Glc(6,1)Glc(6)-Ace |
| 55 | malonyl-ginsenoside $Rb_1$ | Glc(2,1)Glc(6)-Mal | Glc(2,1)Glc |
| 60 | malonyl-ginsenoside Rc | Glc(2,1)Glc(6)-Mal | Glc(6,1)Ara(*f*) |
| 63 | $Rb_2$ | Glc(2,1)Glc | Glc(6,1)Ara(*p*) |
| 65 | $Rb_3$ | Glc(2,1)Glc | Glc(6,1)Xyl |
| 59/67/69/71/76/77/82 | $Rs_1$ | Glc(2,1)Glc(6)-Ace | Glc(6,1)Ara(*p*) |
| 59/67/69/71/76/77/82 | $Rs_2$ | Glc(2,1)Glc(6)-Ace | Glc(6,1)Ara(*f*) |
| 59/67/69/71/76/77/82 | Pseudoginsenoside $F_8$ | Glc-[6-Ace]-(2,1)Glc | Glc(6,1)Ara(*p*) |
| 68 | malonyl-ginsenoside $Rb_2$ | Glc(2,1)Glc(6)-Mal | Glc(6,1)Ara(*p*) |
| 78 | malonyl-ginsenoside $Rb_3$ | Glc(2,1)Glc(6)-Mal | Glc(6,1)Xyl |
| 79 | Rd | Glc(2,1)Glc | Glc |

| | | | |
|---|---|---|---|
| 80/81/86/89/93 | Pseudoginsenoside $Rc_1$ | Glc(2,1)Glc(6)-Ace | Glc |
| 80/81/86/89/93 | Quinquenoside III | Glc-[6-Ace]-(2,1)Glc | Glc |
| 80/81/86/89/93 | β-D-Glucopyranoside, (3β,12β)-3-(β-D-glucopyranosyloxy)-12-hydroxydammar-24-en-20-yl 6-O-(6-O-Acetyl-β-D-glucopyranosyl)- (9CI) | Glc | Glc(6,1)Glc(6)-Ace |
| 85 | Gypenoside XVII | Glc | Glc(6,1)Glc |
| 87 | Ginsenoside Ra6/Quinquenoside II | Glc(2,1)Glc(6)-But | Glc(6,1)Glc |
| 88 | malonyl-ginsenoside Rd | Glc(2,1)Glc(6)-Mal | Glc |
| 91 | Ginsenoside $Ra_7$ | Glc(2,1)Glc(6)-But | Glc(6,1)Ara(*p*) |
| 92 | Gypenoside IX | Glc | Glc(6,1)Xyl |
| 94/98 | Ginsenoside $Ra_8$/Ginsenoside $Ra_9$ | Glc(2,1)Glc(3)-But/Glc(2,1)Glc(6)-But | Glc(6,1)Ara(*f*) |
| 96 | Quinquenoside I | Glc(2,1)Glc(6)-But | Glc |
| 97 | (20S)-Ginsenoside $Rg_3$ | Glc(2,1)Glc | H |
| 99 | 20(S)-Ginsenoside $Rs_3$ | Glc(2,1)Glc(6)-Ace | H |
| 100 | (20R)-Ginsenoside $Rg_3$ | Glc(2,1)Glc | H |
| 101 | (20R)-Ginsenoside $Rs_3$ | Glc(2,1)Glc(6)-Ace | H |
| 103 | 20(S)-Ginsenoside $Rh_2$ | Glc | H |
| 104 | 20(R)-Ginsenoside $Rh_2$ | Glc | H |

Table S1-2. Chemical structures of the detected compounds in different parts of *P. ginseng* root (PPT-type).

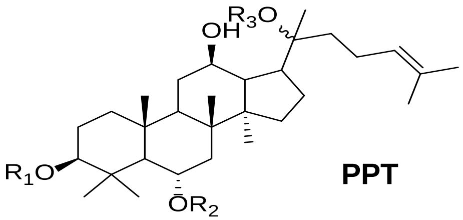


**PPT**

| No | Name | R1 | R2 | R3 |
|---|---|---|---|---|
| 7/11/15 | Ginsenoside $Re_1$ | H | Glc | Glc(3,1)Glc |
| 7/11/15 | Ginsenoside $Re_2$ | H | Glc(3,1)Glc | Glc |
| 7/11/15 | Ginsenoside $Re_3$ | H | Glc | Glc(4,1)Glc |
| 8/10 | Ginsenoside $Re_4$ | H | Glc | Glc(6,1)Ara(f) |
| 9 | 20-O-Glucosylginsenoside Rf | H | Glc(2,1)Glc | Glc |
| 12/17 | Yesanchinoside E | H | Glc(2,1)Rha | Glc(6,1)Glc |
| 13 | Floralginsenoside M/Floralginsenoside N | H | Glc(2,1)Rha | Glc(6,1)Ara(*f*)/Glc(6,1)Ara(*p*) |
| 14 | Notoginsenoside $R_1$ | H | Glc(2,1)Xyl | Glc |
| 18/27/33/36/37 | Vinaginsenoside $R_4$ | Glc(2,1)Glc | H | Glc |
| 18/27/33/36/37 | Notoginsenoside $R_3$ | H | Glc | Glc(6,1)Glc |
| 18/27/33/36/37 | Notoginsenoside $R_6$ | Glc | H | Glc(6,1)Glc |
| 18/27/33/36/37 | Notoginsenoside M | H | Glc(6,1)Glc | Glc |
| 18/27/33/36/37 | Notoginsenoside N | H | Glc(4,1)Glc | Glc |
| 19 | $Rg_1$ | H | Glc | Glc |

| | | | | |
|---|---|---|---|---|
| 20 | Re | H | Glc(2,1)Rha | Glc |
| 21/23/25 | 6'-O-Acetyl-ginsenoside $Rg_1$ | H | Glc(6)-Ace | Glc |
| 22/24 | 6'''-O-Acetyl-ginsenoside Re | H | Glc(2,1)Rha | Glc(6)-Ace |
| 28/31 | Floralginsenoside P | Glc(2,1)Glc | H | Glc(6,1)Ara(*p*) |
| 32/41 | Yesanchinoside D | H | Glc(6)-Ace | Glc |
| 39 | Rf | H | Glc(2,1)Glc | H |
| 40 | Ginsenoside $Re_6$ | H | Glc | Glc(6)-But |
| 40 | Koryoginsenoside $R_1$ | H | Glc(6)-But | Glc |
| 43 | Notoginsenoside $R_2$ | H | Glc(2,1)Xyl | H |
| 46 | $Rg_2$ | H | Glc(2,1)Rha | H |
| 47 | 20(R)-Ginsenoside $Rh_1$ | H | Glc | H |

Table S1-3. Chemical structures of the detected compounds in different parts of *P. ginseng* root (OA-type).

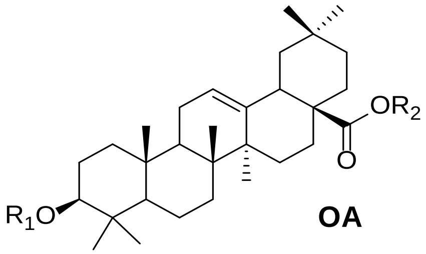


**OA**

| No | Name | R1 | R2 |
|---|---|---|---|
| 53 | Ro | GlurA(2,1)Glc | Glc |
| 75/95 | Chikusetsusaponin Iva | GlurA | Glc |

| | | | |
|---|---|---|---|
| 30 | Pseudoginsenoside $Rt_1$ | GlurA(2,1)Xyl | Glc |

Table S1-4. Chemical structures of the detected compounds in different parts of *P. ginseng* root (others).

| No | Name | Molecular structure |
|---|---|---|
| 1/3/4/34 | Ginsenoside $Re_5$ isomer | 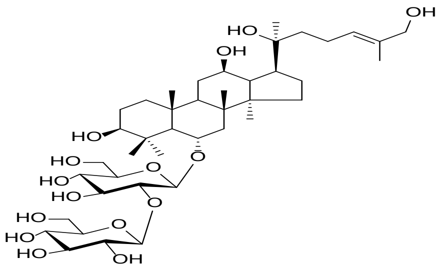 |
| 5 | Koryoginsenoside $R_2$ | 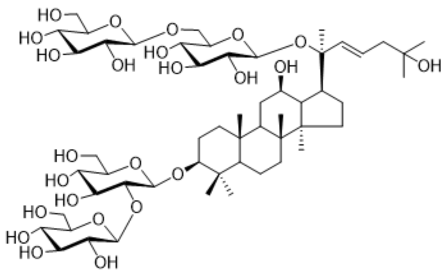 |

| | | |
|---|---|---|
| 5 | Ginsenoside V (24β) | 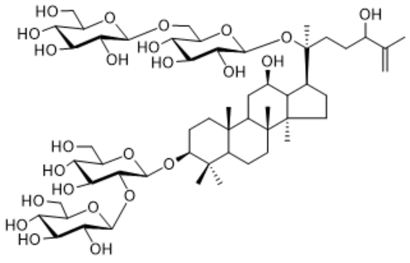 |
| 16 | Notoginsenoside B | 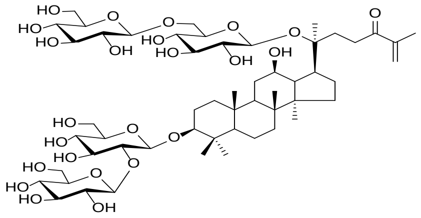 |
| 26 | Ginsenoside $Rg_5$ | 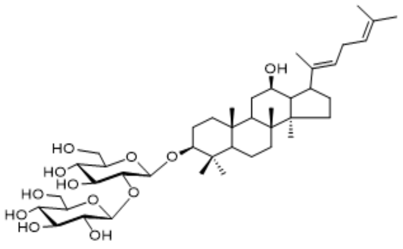 |
| 29 | Ginsenoside $Rs_4$ | 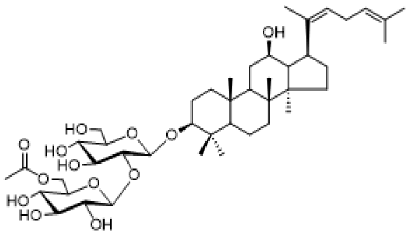 |

| | | |
|---|---|---|
| 29 | Ginsenoside $Rs_5$ | 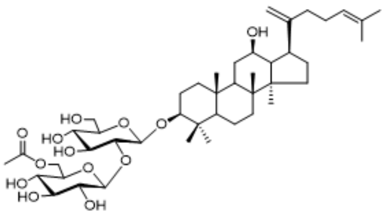 |
| 35 | 12,23-Eproxyginsenoside $Rg_1$ | 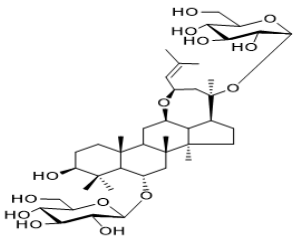 |
| 102/105 | Ginsenoside $Rk_1$ | 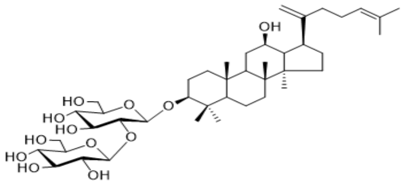 |

| | | |
|---|---|---|
| 102/105 | Ginsenoside $Rz_1$ | 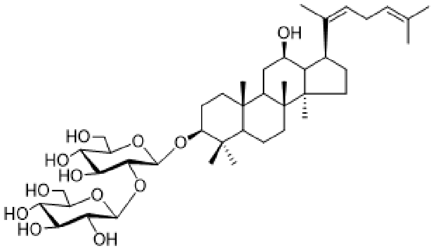 |

Table S2 Detailed information of the tested *P. ginseng* whole root samples.

| Sample number | Source | Growth year | Collection Time |
|---|---|---|---|
| HLJ-1 | Sanjitai forest farm, Suiling County, Heilongjiang Province, China | 4 | 2019.10–2019.11 |
| HLJ-2 | Sanjitai forest farm, Suiling County, Heilongjiang Province, China | 4 | 2019.10–2019.11 |
| HLJ-3 | Sanjitai forest farm, Suiling County, Heilongjiang Province, China | 4 | 2019.10–2019.11 |
| HLJ-4 | Shuguang forest farm, Tieli City, Heilongjiang Province, China | 4 | 2019.10–2019.11 |
| HLJ-5 | Shuguang forest farm, Tieli City, Heilongjiang Province, China | 4 | 2019.10–2019.11 |
| HLJ-6 | Shuguang forest farm, Tieli City, Heilongjiang Province, China | 4 | 2019.10–2019.11 |
| HLJ-7 | Baoqing County, Shuangyashan City, Heilongjiang Province, China | 5 | 2019.10–2019.11 |
| HLJ-8 | Baoqing County, Shuangyashan City, Heilongjiang Province, China | 5 | 2019.10–2019.11 |

| | | | |
|---|---|---|---|
| HLJ-9 | Baoqing County, Shuangyashan City, Heilongjiang Province, China | 5 | 2019.10–2019.11 |
| JL-1 | Tonghua City, Jilin Province, China | 4 | 2019.10–2019.11 |
| JL-2 | Tonghua City, Jilin Province, China | 4 | 2019.10–2019.11 |
| JL-3 | Tonghua City, Jilin Province, China | 4 | 2019.10–2019.11 |
| JL-4 | Taishang Town, Ji'an City, Jilin Province, China | 4 | 2019.10–2019.11 |
| JL-5 | Taishang Town, Ji'an City, Jilin Province, China | 4 | 2019.10–2019.11 |
| JL-6 | Taishang Town, Ji'an City, Jilin Province, China | 4 | 2019.10–2019.11 |
| JL-7 | Taishang Town, Ji'an City, Jilin Province, China | 4 | 2019.10–2019.11 |
| JL-8 | Taishang Town, Ji'an City, Jilin Province, China | 4 | 2019.10–2019.11 |
| JL-9 | Taishang Town, Ji'an City, Jilin Province, China | 4 | 2019.10–2019.11 |
| JL-10 | Jiayi Village, Qinghe Town, Ji'an City, Jilin Province, China | 5 | 2019.10–2019.11 |
| JL-11 | Jiayi Village, Qinghe Town, Ji'an City, Jilin Province, China | 5 | 2019.10–2019.11 |
| JL-12 | Jiayi Village, Qinghe Town, Ji'an City, Jilin Province, China | 5 | 2019.10–2019.11 |
| JL-13 | Xintuntun Village, Xintun Town, Fusong County, Jilin Province, China | 5 | 2019.10–2019.11 |
| JL-14 | Xintuntun Village, Xintun Town, Fusong County, Jilin Province, China | 5 | 2019.10–2019.11 |

| | | | |
|---|---|---|---|
| JL-15 | Xintuntun Village, Xintun Town, Fusong County, Jilin Province, China | 5 | 2019.10–2019.11 |
| JL-16 | Quanyang Town, Fusong County, Jilin Province, China | 5 | 2019.10–2019.11 |
| JL-17 | Quanyang Town, Fusong County, Jilin Province, China | 5 | 2019.10–2019.11 |
| JL-18 | Quanyang Town, Fusong County, Jilin Province, China | 5 | 2019.10–2019.11 |
| LN-1 | Yongling Town, Xinbin County, Fushun City, Liaoning Province, China | 4 | 2019.10–2019.11 |
| LN-2 | Yongling Town, Xinbin County, Fushun City, Liaoning Province, China | 4 | 2019.10–2019.11 |
| LN-3 | Yongling Town, Xinbin County, Fushun City, Liaoning Province, China | 4 | 2019.10–2019.11 |
| LN-4 | Dachuantou Town, Kuandian County, Dandong City, Liaoning Province, China | 4 | 2019.10–2019.11 |
| LN-5 | Dachuantou Town, Kuandian County, Dandong City, Liaoning Province, China | 4 | 2019.10–2019.11 |
| LN-6 | Dachuantou Town, Kuandian County, Dandong City, Liaoning Province, China | 4 | 2019.10–2019.11 |
| LN-7 | Shizhuzi Village, Kuandian County, Dandong City, Liaoning Province, China | 5 | 2019.10–2019.11 |
| LN-8 | Shizhuzi Village, Kuandian County, Dandong City, Liaoning Province, China | 5 | 2019.10–2019.11 |
| LN-9 | Shizhuzi Village, Kuandian County, Dandong City, Liaoning Province, China | 5 | 2019.10–2019.11 |
